# Treatment of Femoropopliteal Artery Disease with Polymer-Coated Drug-Eluting Stent: 5-Year Results of a Prospective, Non-Randomized Study Including the Halo Phenomenon

**DOI:** 10.1007/s00270-023-03652-2

**Published:** 2024-01-16

**Authors:** Giovanni Federico Torsello, Konstantinos Stavroulakis, Theodosios Bisdas, Yamel Cardona, Katrin Wichmann, Giovanni Battista Torsello

**Affiliations:** 1 Department of Diagnostic and Interventional Radiology, Institute of Diagnostic and Interventional Radiology, Göttingen Medical Center, University Medical Center Göttingen, Robert-Koch-Strasse 40, 37075 Göttingen, Germany; 2 Department of Vascular Surgery, Ludwigs-Maximilians-University, Munich, Germany; 3 Department of Vascular Surgery, Athens Medical Center, Athens, Greece; 4 Institute for Vascular Research, St. Franziskus Hospital, Münster, Germany

**Keywords:** Drug-eluting stent, Paclitaxel, Peripheral arterial disease, Femoropopliteal artery, Halo sign

## Abstract

**Purpose:**

To investigate the long-term results of the Eluvia drug-eluting stent (DES) implantation for femoropopliteal arterial disease, including the ‘halo’ phenomenon. Long-term data of DES is scarce. A focal reaction (‘halo’) following Eluvia DES deployment has been described. However, the long-term clinical impact of this phenomenon remains unclear.

**Methods:**

This prospective, non-randomized, single-arm study included 130 consecutive patients treated with an Eluvia DES for symptomatic femoropopliteal disease between March 2016 and December 2018. Clinical outcomes and imaging were assessed after 6 months and annually thereafter for up to 5 years. The primary outcome measure was primary patency. Secondary outcomes were freedom from clinically driven target lesion revascularization (CD-TLR), freedom from major amputation, overall survival and amputation-free survival rates.

**Results:**

The primary patency was 65% at 5 years. The freedom from CD-TLR and from major amputation at 5 years was 79 and 96%, respectively. The overall survival and amputation-free survival rates were 88 and 83% at 60 months, respectively. Out of the 27 patients with a halo sign, two showed an increased (7.4%) and 6 (22.2%) a decreased diameter. In 19 cases (70.4%), the diameter remained unchanged at the latest follow-up. The presence of the ‘halo’ sign was associated with increased primary patency (87% versus 59%, HR: 2.48, 95%CI 1.19–5.16, *P* = .015).

**Conclusions:**

The presented patient cohort treated with the Eluvia DES for femoropopliteal artery lesions indicates durable efficacy and a good safety profile regardless of the halo phenomenon. The results need to be confirmed in a larger patient cohort.

**Level of Evidence III:**

Non-randomized controlled cohort/follow-up study.

**Graphical Abstract:**

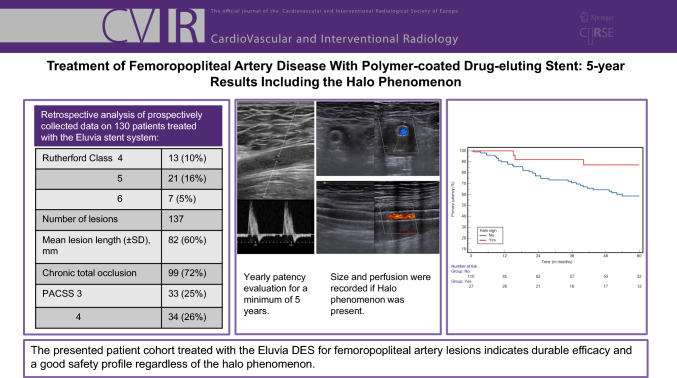

## Introduction

Endovascular treatment is the first treatment option for most patients with symptomatic peripheral arterial disease [1]. Although percutaneous procedures are beneficial because of their minimally invasive nature, restenosis, clinical failure and repeated interventions are not rare after endovascular repair [2].

Paclitaxel can be delivered in the vessel wall during the index procedure with either drug-coated balloons or drug-eluting stents (DES) in an effort to inhibit the development of intimal hyperplasia. Although drug-coated balloons have been shown to be effective in treating short, non-heavily calcified diseases, their performance in complex lesions is unclear [3]. On the contrary, pragmatic cohorts showed promising results with the use of the polymer-coated Eluvia DES (Boston Scientific, Marlborough, MA, USA) for challenging lesions [4], while randomized evidence revealed superior primary patency vs bare metal stent at 1 year [5] and vs. polymer-free drug-coated stent at 2 years [6]. Nonetheless, it remains unknown if the observed ‘halo’ sign, which is frequently observed following the deployment of the Eluvia scaffold, has an impact on the long-term performance of this platform [4, 7, 8]. It is hypothesized that prolonged paclitaxel elution might lead to a vessel wall degeneration with malapposition of the stent and late thrombosis, as observed in the coronaries [4]. Furthermore, there is currently no long-term data of the Eluvia DES in the current body of evidence. The purpose of this study was to evaluate the long-term outcome of patients treated with the Eluvia DES and in particular of the patients showing a ‘halo’ sign during the first 2 years after implantation.

## Methods

### Study Design

This is a single-center, single-arm, retrospective analysis of prospectively collected data. It continues an analysis that was previously published, maintaining patient population, study design and follow-up protocols, seeking to provide long-term data on that previously described patient population. Approval from Ethics Committee was obtained to conduct the evaluation (N:2021-220-f-S). The study was performed in accordance with the principles of the Declaration of Helsinki, and written informed consent was required from every patient prior to procedure.

The authors had full access to all the data in the study and take responsibility for its integrity and the data analysis.

### Study Population

Between March 2016 and December 2018, all patients treated with the Eluvia DES for symptomatic femoropopliteal disease were included in this analysis. Inclusion criteria for the study included: presentation with Rutherford category 2 to 6 symptomatology, lesions in the native superficial femoral artery (SFA) and/or popliteal artery with chronic total occlusion or stenosis ≥ 70% by angiographic assessment, recoil or flow-limiting dissection after plain old balloon angioplasty (POBA).

Exclusion criteria were in-stent-restenosis, aneurysmatic degeneration of the target lesion prior to treatment, common/deep femoral artery stenosis, or bypass anastomosis stenosis.

The 2 year results of this patient cohort has previously been published [4]. This analysis was conducted to be able to determine the long-term results of that patient population.

### Study Device

The interventional device was the Eluvia DES designed for the treatment of lesions in the femoropopliteal arteries and commercially available in Europe since February 2016. The Eluvia stent is deployed on a 6-F triaxial delivery system. The stent utilizes a low-dose anti-restenotic drug paclitaxel (0.167 μg/mm^2^) in conjunction with a biocompatible fluoropolymer. This drug and polymer combination is designed to sustain drug release for more than 1 year to match the timing of restenosis in the femoropopliteal arteries.

### Procedure and Follow-up

Patient demographics and comorbidities as well as clinical data were prospectively collected. In the previous evaluation, a high rate of vessel wall degeneration was observed after 2 years. Hence, during the successive follow-up visits, we took special care to evaluate those patients with this phenomenon while maintaining the original scientific and clinical workup. All patients were treated in local anesthesia and lower extremity angiographic assessment was performed with standard techniques. The angiography units used were both Artis zee ceiling (Siemens, Erlangen, Germany). Anticoagulant therapy consistent with hospital standards was employed during stenting procedures. The main indication for the deployment of the scaffold was presence of recoil or flow-limiting dissection following POBA. In case of thrombotic lesions, endovascular thrombectomy was performed. Stents were sized according to the instructions for use. In case of more than one stent deployment, the maximum overlap between the stents was 10 mm. Post-dilatation was performed at the investigator’s discretion. Iliac artery lesions were allowed to be treated during the index procedure prior to target lesion treatment.

Dual antiplatelet therapy following implantation was required for a minimum of 3 months followed by 100 mg/d aspirin or 75 mg/d clopidogrel monotherapy lifelong. Patients under oral anticoagulants maintained their medication with an additional antiplatelet therapy for 3 months after index procedure.

Follow-up examinations were scheduled at 6 and 12 months and then yearly post-index procedure. In these visits, clinical and hemodynamic characteristics were assessed. Patency of the treated vessels was assessed by duplex ultrasound (DUS) at each follow-up with Acuson systems (Siemens). In case of clinical worsening, a CT angiography (CTA) or conventional angiography was performed additionally. The CT scanner used was a Somatom Definition AS (Siemens). Any available conventional angiograms and CTA images were reviewed using JiveX (VISUS, Bochum, Germany).

Additional B-mode duplex ultrasound of the stented arteries in transverse and longitudinal planes were employed to screen for the halo phenomenon. When applicable, outer-to-outer degenerative arterial wall (‘halo’) dimensions were measured and recorded.

### Outcome Measures

The primary outcome measure of this study was primary patency. Secondary outcomes were freedom from clinically driven target lesion revascularization (CD-TLR), freedom from major amputation, overall survival and amputation-free survival rate. Additionally, the outcomes of arterial wall degeneration as well as clinical and hemodynamic improvement at 5 years were assessed.

### Definitions

Primary patency was defined as duplex ultrasound peak systolic velocity ratio ≤ 2.0 in the absence of CD-TLR or bypass surgery. The measurement of peak systolic velocity was performed in the lesion and 1 cm proximal to the lesion.

CD-TLR was defined as any reintervention within 5 mm proximal or distal to the originally treated segment for > 50% stenosis in the presence of recurrent symptoms. Major amputation was defined as any above-ankle amputation. As the prolonged paclitaxel elution might lead to a focal reaction with vessel wall degeneration at the level of the stent (‘Halo’), this segment was evaluated by Duplex ultrasound. According to the literature [4, 7, 8], the Halo sign was defined as an increase in the diameter of the treated segment of ≥ 50% compared to the non-treated vessel segment with or without blood flow between the stent and the vessel wall. Secondary outcomes included clinical improvement, defined as a decrease in Rutherford classification by one or more categories compared to baseline. The degree of calcification was graded on the basis of the Peripheral Arterial Calcium Scoring Scale (PACSS) classification [9].

### Statistical Analysis

For the statistical analysis and graphics, the MedCalc Statistical Software (version 12.4.0.0; MedCalc Software, Ostend, Belgium) was used. Continuous variables are presented as means ± standard deviation or median (Interquartile range), while categorical data are given as counts. Continuous numeric variables were compared by Student’s t-test for paired samples or the Wilcoxon test according to their distribution (determined by D’Agostino-Pearson test). Cumulative primary and secondary patency, as well as freedom from CD-TLR and amputation-free-survival were estimated using the Kaplan–Meier method and compared by the log-rank test between the group of claudicants and patients with chronic limb-threatening ischemia (CLTI). The Kaplan–Meier curves were truncated when the standard error exceeded 10%. A Cox regression analysis to identify risk factors (chronic limb-threatening ischemia, chronic total occlusion, PACCS 3 or 4, lesion length > 250 mm, stenting over P1 segment, “halo” sign) for patency loss during surveillance was performed. The threshold of statistical significance was *p* ≤ 0.05.

### Funding

The previous study was supported with a scientific grant from Boston Scientific. The company was not involved in any of the study elements, including design, data collection or analysis or manuscript creation. After publication of the 2 year results, funding was completed. The present study was not financially supported.

## Results

### Demographics Data and Lesion Characteristics

The baseline characteristics of the 130 consecutive subjects enrolled in the study had previously been described in an earlier study on the 2 year results [4]. Briefly, mean age was 71 ± 8 years, 82 (63%) were male, 35% had a history of coronary artery disease and/or diabetes mellitus, 13% previous limb revascularization, and 31% suffered from CLTI. Preoperative evaluation included duplex ultrasound in all patients, CT angiography in 122 and MR angiography in 8.137 lesions (74% chronic total occlusions, CTO) with a mean lesion length of 19.4 ± 11 cm were treated. 67% of the lesions were PACSS class 3 or 4.

### Outcomes (Entire Cohort)

**Fig. 1:**
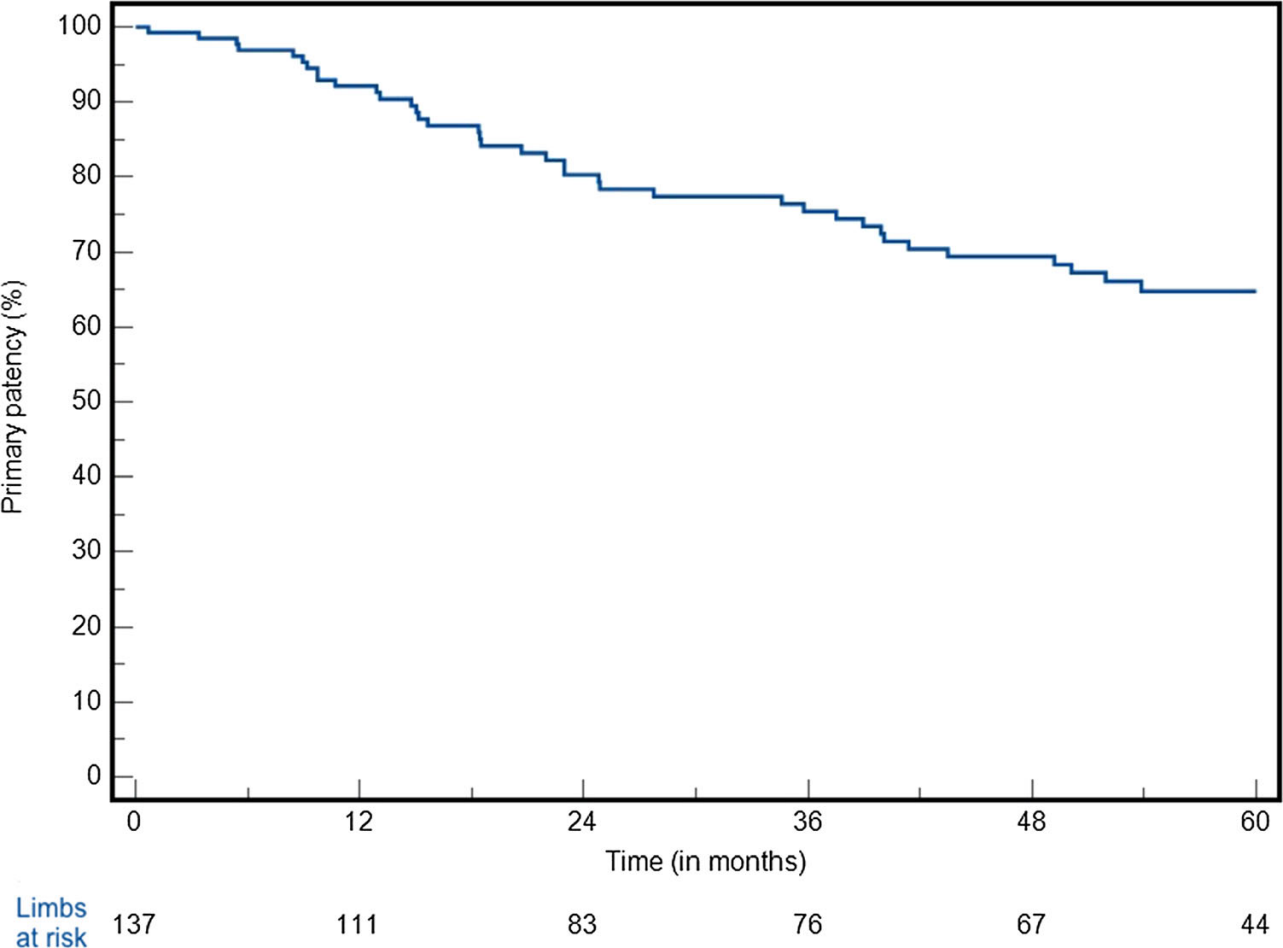
Primary patency at 5 years (SE > 10% after 65 months)

**Fig. 2:**
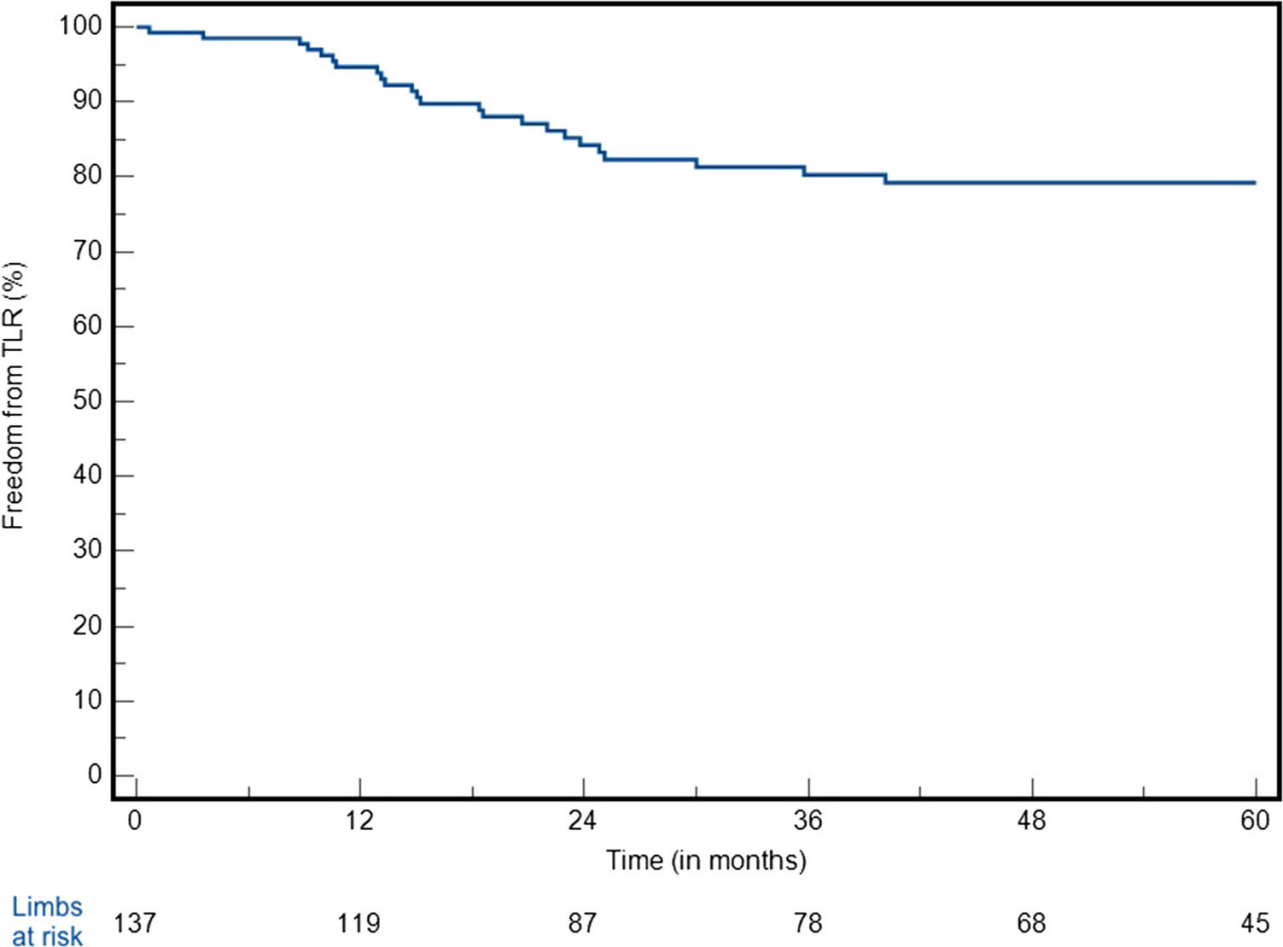
Freedom from CD-TLR at 5 years (SE > 10% after 79.5 months)

**Fig. 3:**
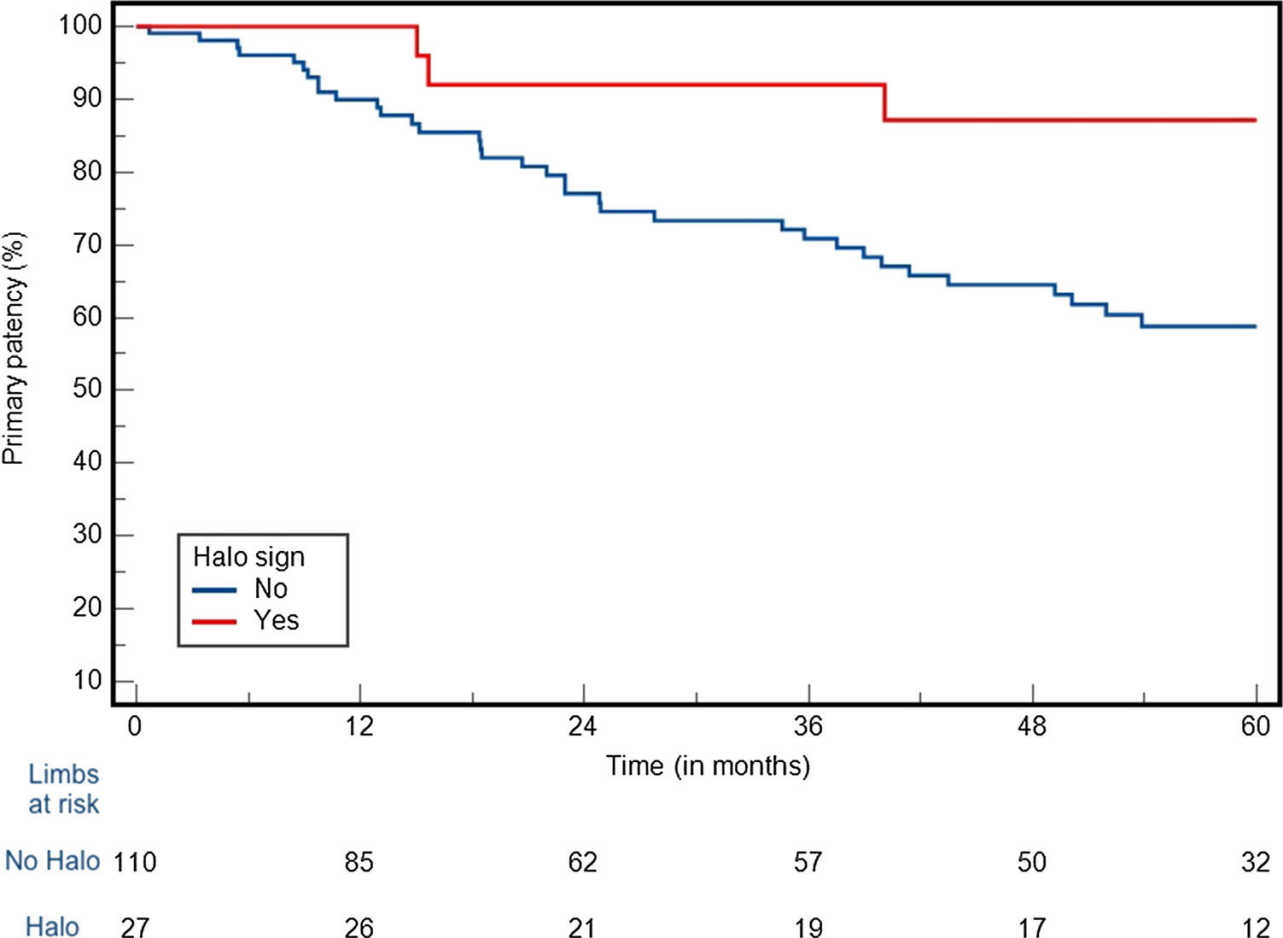
Primary patency at 5 years between patients with and without halo sign (SE > 10% 65 months for both groups)

The median follow-up was 60 months (IQR 23 to 70 months). At 5 years, the primary patency rate was 65% (Fig. 1). The freedom from CD-TLR at 5 years was 79% (Fig. 2), the freedom from major amputation at 5 years was 96%, respectively. At 5 years, amputation-free survival was 83%. Fifteen patients (11%) died during the follow-up. Regarding the clinical status of the remaining study cohort at the last follow-up, the majority of patients (*n* = 121 patients, 88%) were either asymptomatic or had mild claudication (class 1 or 2), 7 patients (5%) had severe claudication (class 3), while 9 patients (7%) had persistent tissue loss (class 5). The Cox-regression analysis of different anatomic factors for primary patency (chronic limb-threatening ischemia, chronic total occlusion, PACCS 3 or 4, lesion length > 250 mm, stenting in the popliteal segment, the’halo’ sign) showed a protective effect of “halo” sign (HR: 0.24, 95%CI 0.07–0.79, *P* = 0.019, Table 1) (Fig. 3).

**Table 1:** Cox-regression analysis for primary patency

Risk factors	HR	95% CI	*P* value
CLTI CTO PACCS 3 or 4 Lesion length > 250 mm Stenting over P1 “Halo” sign	1.48 2.20 0.75 1.5 1.28 0.24	0.75 to 2.96 0.82 to 5.95 0.39 to 1.44 0.78 to 2.92 0.67 to 2.44 0.07 to 0.79	0.261 0.119 0.391 0.222 0.461 0.019

*CLTI* chronic limb-threatening ischemia, *CTO* chronic total occlusion, *PACCS* peripheral arterial calcium scoring system

### Outcome of Patients with the ‘Halo’

At 2 years, the ‘halo’ phenomenon was observed in 20% (*n* = 27) of the patients. Mean lesion length was 219.23 ± 91.8 mm. All lesions were CTO and had to be treated with different additional techniques: Thrombectomy (*n* = 5), atherectomy (*n* = 2), scoring balloon (*n* = 1), subintimal recanalization utilizing an Outback catheter (*n* = 1), and distal retrograde access (*n* = 1).

**Fig. 4:**
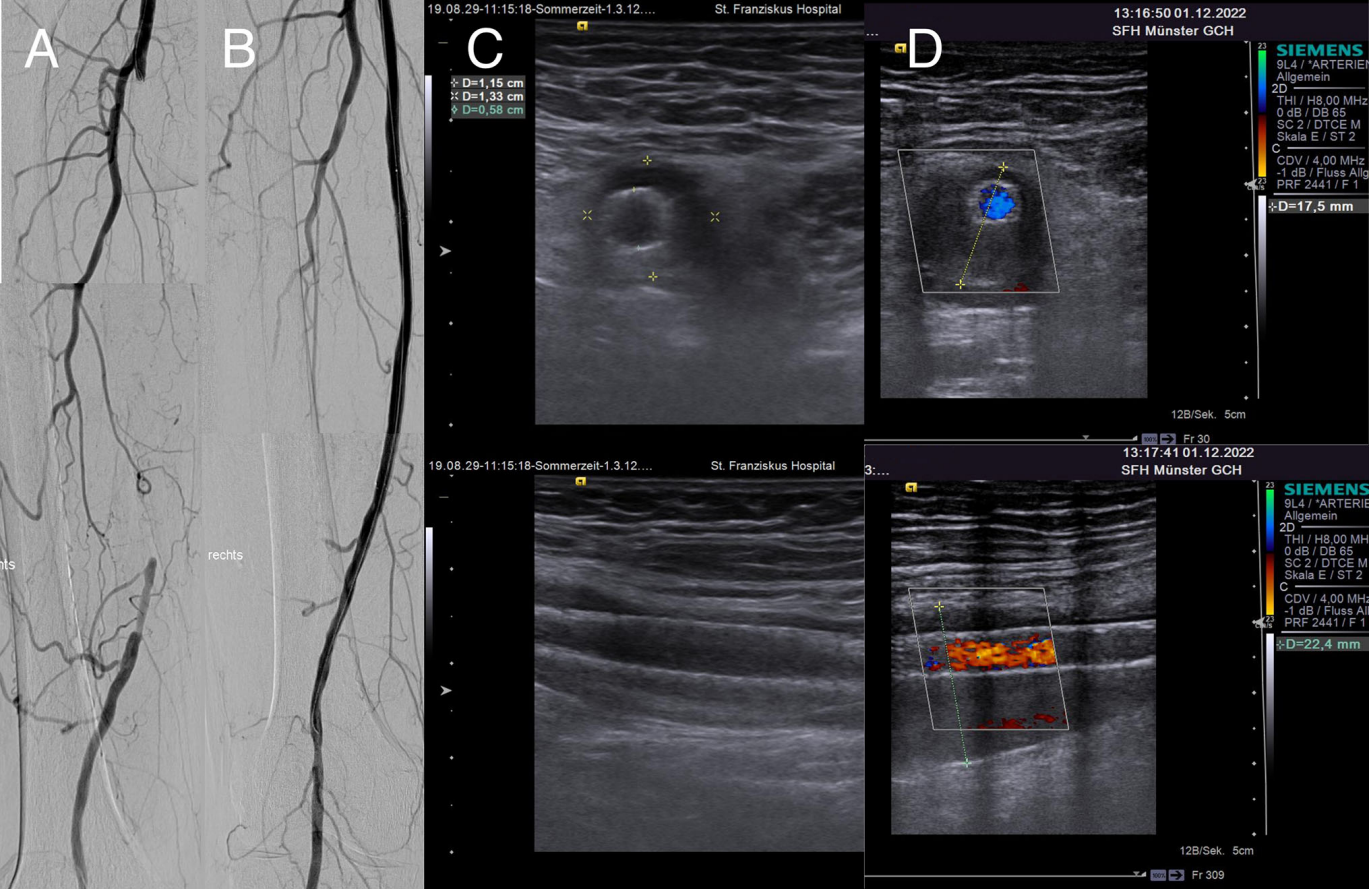
Halo phenomenon after Eluvia deployment (Central Illustration). **A** Angiogram before recanalization of a chronic total occlusion (CTO) of the superficial femoral artery (SFA) of the right limb. **B** Final angiogram following Eluvia DES deployment. **C** Ultrasound follow-up 16 months after initial deployment revealed ahalo phenomenon. In the transverse plane, a maximum diameter of 13 mm is measured. The stent is patent at that time. **D** Duplex ultrasound 56 months after Eluvia deployment, the maximum diameter increased to at least 18 mm in the transverse plane

Two patients (7.4%) with a ‘halo’ phenomenon passed away, 31 and 40 months after the treatment. Both deaths were unrelated to the stent or procedure (sepsis and mesenteric ischemia, respectively). At the latest follow-up, duplex ultrasound showed an increased halo diameter in two cases (7.4%), a decrease in 6 (22.2%), and unchanged in the remaining 19 lesions (70.4%) (Fig. 4, Central Illustration). During the follow-up, CD-TLR was necessary in 5 cases (18.5%).

At 5 years, primary patency was 87% for those with halo sign and 59% for those without a ‘halo’ sign (HR:2.48, 95%CI: 1.19 to 5.16, *P* = 0.015).

## Discussion

### Clinical Evidence of Polymer-Coated Drug-Eluting Stents

The present study evaluates the long-term outcomes of the polymer-coated Eluvia DES for the treatment of femoropopliteal artery lesions in a real-world cohort. Being the first study to report 5 year data on the Eluvia device, two main observations are made in the present single-arm prospective study. Firstly, this study adds to the current body of evidence that the halo phenomenon described in earlier studies had no negative clinical impact, but mostly remained stable. Secondly, despite the challenging patient sample, primary patency and freedom from CD-TLR rates were excellent after 5 years, sustaining patency rates described in shorter-term studies.

The EMINENT study demonstrated superior patency for Eluvia DES compared with BMS in femoropopliteal lesion treatment through 1 year [5]. The 2 year results of the IMPERIAL study demonstrated a significantly lower CD-TLR rate for patients treated with Eluvia compared to the Zilver PTX DES [6]. The Kaplan–Meier estimate of primary patency at 24 months was 83.0% for Eluvia and 77.1% for Zilver PTX.

Our results were obtained in a cohort including patients with multiple comorbidities and complex anatomies. Of note, the mean lesion length was 19.4 cm, 74% of the lesions were CTOs and 67% of the cases were severely calcified. This was also a real-world cohort including patients with CLTI (31%), chronic kidney disease (24%) and end-stage renal disease (5%), after previous endovascular or open surgical treatment (11 and 2%, respectively). In that regard, patient characteristics differed from the otherwise highly selected patient samples in the EMINENT and IMPERIAL trials, e.g., excluding Rutherford categories 4 and 5 as well as chronic kidney disease. This leads to a relevant gap of data regarding the durability of endovascular treatment in real-world cohorts.

CAPSICUM is a multicenter, prospective, observational study evaluating 1,204 limbs (CLTI: 34.8%, mean lesion length: 18.6 cm, CTO: 53.2%, bilateral wall calcification: 41.9%) of 1,097 patients with peripheral artery disease (Diabetes mellitus: 60.8%, chronic kidney disease: 66.2%) [10]. Considering the severity of the disease, the 1 year restenosis rate (12.9%) and TLR rate (6.2%) were acceptable.

In the DESAFINADO registry, 64 patients with 67 femoropopliteal lesions were included; 78% suffered from diabetes and 84% had CLTI. Of those with ischemic wounds, 79% did not have run-off to the foot [11]. Mean lesion length was 193 mm and 52% were severely calcified. Still, primary patency at 1 year was 84% in the overall cohort. Twelve-month freedom from CD-TLR, limb salvage, survival, and amputation-free survival rates were 92%, 93%, 85%, and 80%, respectively. In both registries, the results of Eluvia in CLTI-dominant patient populations were promising but limited to 1 year.

Our data provide evidence for acceptable long-term durability of polymer-coated drug-eluting stents in routine clinical practice and in patients with challenging lesions. However, some authors consider the vessel wall degeneration after the use of fluoropolymer-coated paclitaxel-eluting stents in the superficial femoral artery as a “rising concern” [7].

### Evidence of the Halo Phenomenon

In the 1 year analysis of our cohort [8], we termed the finding on B-mode ultrasound as a “vessel wall degeneration” around the implanted Eluvia to be present in five cases. We observed this phenomenon with a rate of 20% at 2 years in our all-comers registry [4]. The finding was confirmed also in other studies with a prevalence of 33.7% in the IMPERIAL study [6], 16.8% in the CAPSICUM registry [10] and 26.1% in the EMINENT study [5]. In the coronary arteries, a similar halo phenomenon was detected after rotational atherectomy [12].

The pathophysiology of this phenomenon has not been elucidated so far. Based on immunohistology and magnetic resonance imaging, an excessive vessel wall inflammation similar to changes secondary to vasculitis was hypothesized. Experimental studies [13] comparing the vascular healing responses of healthy swine arteries treated with polymer-free or fluoropolymer-based paclitaxel-eluting stent showed that the presence of a permanent polymer may contribute to differential vascular responses like medial layer disruption and aneurysmal vessel degeneration.

However, halo signs are not specific to the Eluvia stent. Similar findings were described also after the use of bare metal stents [5, 14], stent grafts [14], Zilver PTX polymer-free drug-coated stents [4, 6], and drug-coated balloons following directional atherectomy [15] for treatment of femoropopliteal lesions. All mentioned treatment modalities induce a measure of vascular inflammatory response.

In our study, halo phenomena were observed more frequently in patients with severe dissection after PTA, mechanical thrombectomy, atherectomy and additional use of DCB. In CAPSICUM [10], subintimal wire passage was significantly associated with an increased observation of the halo sign at 1 year, which the investigators termed as “aneurysmal degeneration.” Of note, also intravascular ultrasound (IVUS) use was found to be a risk factor for the appearance of halos. While the exact causality is unclear, it might be hypothesized that IVUS-based measurements leads to oversizing of endovascular devices. This consideration suggests that also the severity of vessel wall damage during the procedure could play an important role in local inflammation and thus halo development.

Lastly, the clinical impact of the halo phenomenon must be established. Blood flow outside the stent was initially observed only in two patients secondary to reintervention for stent occlusion including fibrinolysis. Secondly, during the 5-year follow-up diameter increase was observed only in two cases. No patient experienced rupture, peripheral embolization or occlusion during the remaining 3 years of follow-up. Two patients died for unrelated causes. Thus, the present evaluation suggests that halos seem to be a finding that does not appear to increase the risk of morbidity and mortality after Eluvia implantation. On the contrary, primary patency was higher in patients with a halo compared to those without at 5 years. This observation could be a result of an increased local effect of paclitaxel.

### Limitations

A limitation of this study is the single-arm, non-randomized design which does not allow comparisons with other treatment options. However, the study cohort reflects the typical population treated for complex femoropopliteal disease without excluding patients with severe comorbidities. The high heterogeneity of patients and lesions treated explains the variability of the results observed during the follow-up.

## Conclusions

In summary, the use of polymer-coated drug-eluting stents for the treatment of femoropopliteal lesions demonstrates a good safety profile with sustained efficacy over 5 years of follow-up. Late CD-TLR beyond 2 years were rare. The halo phenomenon is a frequent finding with no evidence of negative clinical effect at 5 years.
